# HPV vaccines and cancer prevention, science versus activism

**DOI:** 10.1186/1750-9378-8-6

**Published:** 2013-02-01

**Authors:** Lucija Tomljenovic, Judy Wilyman, Eva Vanamee, Toni Bark, Christopher A Shaw

**Affiliations:** 1 Neural Dynamics Research Group, Vancouver General Hospital Research Pavilion, University of British Columbia, 828 W. 10th Ave, Vancouver, BC, V5Z 1L8, Canada; 2 School of Social Sciences, Media and Communication, University of Wollongong, Wollongong, 2522, Australia; 3 Department of Structural and Chemical Biology, Mount Sinai School of Medicine, 1425 Madison Ave., Rm 1623, New York, NY, 10029, USA; 4 School of Public Health-Healthcare Emergency Management, Boston University, Boston, MA, 02118, USA

## Abstract

The rationale behind current worldwide human papilloma virus (HPV) vaccination programs starts from two basic premises, 1) that HPV vaccines will prevent cervical cancers and save lives and, 2) have no risk of serious side effects. Therefore, efforts should be made to get as many pre-adolescent girls vaccinated in order to decrease the burden of cervical cancer. Careful analysis of HPV vaccine pre- and post-licensure data shows however that both of these premises are at odds with factual evidence and are largely derived from significant misinterpretation of available data.

## Letter

The recent Editorial by Silvia de Sanjosé* [1] is problematic from a variety of perspectives. Mainly, it attempts to portray a complex issue as a simple dichotomy between supposedly unjustified “anti-HPV vaccine activism” and alleged absolute science which has presumably provided indisputable evidence on HPV vaccine safety and efficacy.

In spite of much unwarranted and premature optimism, the fact is however that HPV vaccines have not thus far prevented a single case of cervical cancer (let alone cervical cancer death). Instead, what the clinical trials have shown is that HPV vaccines can prevent some of the pre-cancerous CIN 2/3 lesions associated with HPV-16 and HPV-18 infection, a large fraction of which would spontaneously resolve *regardless* of the vaccination status [2-4]. For example, in adolescent women aged 13 to 24 years, 38% of CIN 2 resolve after one year, 63% after two and 68% after three years [5]. Moreover, the validity of CIN 2 being a cancer precursor is questionable due to high misclassification rates and poor intra- and inter-observer reproducibility in diagnosis, as well as high regression rates [6-9]. According to Castle *et al*. [7] CIN 2 is the least reproducible of all histopathologic diagnoses and may in part reflect sampling error. While CIN 3 is a more reliable marker for cancer progression than CIN 2, the use of this marker is not without caveats [2,10].

Indeed, the optimistic assumption that HPV vaccination (even *if* proven effective against cervical cancer as claimed), will result in 70% reduction of cervical cancers appears to be largely based on premature, exaggerated and invalid surrogate marker-based extrapolations [2,11]. Crucially, these assumptions failed to take into account several important real-world factors such as:

(1) reliability of surrogate-markers (i.e., whether they can accurately measure what they are purport to measure);

(2) efficacy against oncogenic HPV strains not covered by the vaccine;

(3) possibility of increased frequency of infections with these types;

(4) efficacy in women acquiring multiple HPV types;

(5) effects in women with pre-existing HPV infections

It is also noteworthy that Merck’s HPV vaccine Gardasil received priority *Fast* Track approval by the U.S. Food and Drug Administration (FDA) after a 6-month review process, despite the fact that it failed (and still continues to fail) to meet a single one of the four criteria required by the FDA for *Fast Track* approval. Gardasil is demonstrably neither safer nor more effective than Pap screening combined with the loop electrosurgical excision procedure (LEEP) in preventing cervical cancers, nor can it improve the diagnosis of serious cervical cancer outcomes [12]. In this regard, Gerhardus and Razum have recently noted that the “…*unwarranted confidence in the newHPVvaccines led to the impression that there was no need to actually evaluate their effectiveness*” [11].

Similarly, the notion that HPV vaccines have an impressive safety profile can only be supported by highly flawed design of safety trials [2,13] and is contrary to accumulating evidence from vaccine safety surveillance databases and case reports which continue to link HPV vaccination to serious adverse outcomes (including death and permanent disabilities) [2,4,14]. For example, compared to all other vaccines in the U.S. vaccination schedule, Gardasil alone is associated with 61% of all serious adverse reactions (including 63.8% of all deaths and 81.2% cases of permanent disability) in females younger than 30 years of age [12].

Although a report to a vaccine safety surveillance system does not by itself prove that the vaccine caused an adverse reaction, the unusually high frequency of adverse reactions related to HPV vaccines reported worldwide, as well as their consistent pattern (i.e. nervous system-related disorders rank the highest in frequency), points to a potentially causal relationship [2]. Furthermore, matching the data from vaccine surveillance databases is an increasing number of case reports documenting similar serious adverse reactions associated with HPV vaccine administration, with nervous system and autoimmune disorders being the most frequently reported in the medical literature [15-24].

In summary, the optimistic claims that HPV vaccines will prevent cervical cancers and save lives, and that they are extremely safe, rest on assumptions which are misinterpreted and presented to the public as factual evidence. We thus conclude that further reduction of cervical cancers might be best achieved by optimizing cervical screening (which carries no serious health risks) and targeting other factors of the disease rather than by the reliance on vaccines with questionable efficacy and safety profiles [2,25].

To those who wish to promote HPV vaccination as a means for reducing cervical cancer burden, perhaps the following should be asked:

1. HPV vaccines have not been demonstrated to prevent any cervical cancers so why are they being promoted as cervical cancer vaccines?

2. If the majority of HPV infections and a great proportion of pre-cancerous lesions clear spontaneously and without medical treatment and are thus not a reliable indication of cancer later in life, then how can these end-points be used as a reliable indicator of the number of cervical cancer cases that will be prevented by HPV vaccines?

3. How can the clinical trials make an accurate estimate of the risk associated with HPV-vaccines if they are methodologically biased to produce type-2 errors (false negatives [2,4,13])?

4. Can a passive monitoring system such as that used by most vaccine surveillance systems world-wide allow the medical regulatory agencies to make accurate estimates on the real frequency of HPV-vaccine related adverse reactions?

5. Can an accurate estimate of the real frequency of HPV-vaccine related adverse reactions be made if appropriate follow-up and thorough investigation of suspected vaccine related ADRs is not conducted but instead, these cases are a-priori dismissed as being unrelated to the vaccine?

6. Why are women not informed of the fact that in some circumstances (i.e., prior exposure to vaccine-targeted and non-targeted HPV types), HPV vaccination may accelerate the progression of cervical abnormalities [4,26-28]?

7. How can women make a fully informed decision about whether or not to consent to vaccination if crucial information regarding HPV vaccine efficacy and safety is not being disclosed to them?

8. Should the medical health regulators and authorities rely solely on data provided by the vaccine manufacturers to make vaccine-policy decisions and recommendations [12,29]?

## Competing interests

The authors declare that they have no conflict of interests.

## Authors’ contributions

LT was involved in choosing the topic and drafting the initial manuscript. CAS, JW, EV and TB were involved in critically revising the manuscript and additional content. The authors have read and approved the manuscript. This work was supported by the Dwoskin and Katlyn Fox Family Foundations.
